# 

**Published:** 1895-08-24

**Authors:** 


The Treatment of Heart Disease. By Theodore Fisher, M.D.Lond. - 355

Birmingham Throat and Ear Hospital - 355

Medical Progress and Hospital Clinics—

Traumatic Stricture of the Urethra Treated by Resection - 359

Progress in Medicine.—Graves' Disease - 359

Progress in Surgery.—Surgery of the Spine and Cord - 361

Periscope of Dermatology - 363

The Metric System - ?

Annotations.—"Jerry-built" Houses and Diphtheria — The Tottenham Hospital Dispute—The Doctor's Verdict - 357

Notes and News - 358

The Institutional Workshop—

Hospital Construction.—The Parsee Lying-in Hospital, Bombay - 365

Practical Departments.—Poison Bottles - 366

The Story of the Insane from Year to Year - 367

Scraps and Gleanings - 367

The Student of Medicine - 368

### EXTRA SUPPLEMENT.—THE NURSING MIRROR.

News from the Nursing World—Eminently Practical—Additions and Improvements—"Left Till Called For"—A Successful Bazaar—Girl Nursemaids—District Nurses for Solihull—The Royal British Nurses' Association—A Trained Nurse for Dunmow—Precautions Rescinded—On the Labrador—Lepers in the East—The New Nurse—Short Items - cxli

Dundee Royal Infirmary - cxliii

Elementary Physiology for Nurses. By C. F. Marshall, M.D., B.Sc., F.R.C.S. III.—The Circulatory System - cxliii

Hints for Home Nursing - cxliv

Flat Foot Occurring in Nurses - cxliv

Rational Dress in Russia - cxliv

Holidays and Health - cxlv

Everybody's Opinion - cxlv

A Nurse's Winter in Canada—Notes and Queries - cxlvi

